# Challenges Related to Surgical Site Infection Prevention—Results after Standardized Bundle Implementation

**DOI:** 10.3390/jcm10194524

**Published:** 2021-09-29

**Authors:** Jonas Jurt, Martin Hübner, Daniel Clerc, Pauline Curchod, Mohamed A. Abd El Aziz, Dieter Hahnloser, Laurence Senn, Nicolas Demartines, Fabian Grass

**Affiliations:** 1 Department of Visceral Surgery, Lausanne University Hospital CHUV, 1011 Lausanne, Switzerland; jonas.jurt@chuv.ch (J.J.); martin.hubner@chuv.ch (M.H.); daniel.clerc@chuv.ch (D.C.); pauline.curchod@chuv.ch (P.C.); dieter.hahnloser@chuv.ch (D.H.); fabian.grass@chuv.ch (F.G.); 2 Division of Colon and Rectal Surgery, Mayo Clinic, Rochester, MN 55905, USA; mohamad102365@fmed.bu.edu.eg; 3 Department of Hospital Preventive Medicine, Lausanne University Hospital CHUV, 1011 Lausanne, Switzerland; Laurence.Senn@chuv.ch

**Keywords:** surgical site infection, colorectal, bundle, compliance

## Abstract

Aim: The aim of this study was to assess the implementation of an intraoperative standardized surgical site infection (SSI) prevention bundle. Methods: The multimodal, evidence-based care bundle included nine intraoperative items (antibiotic type, timing, and re-dosing; disinfection; induction temperature control > 36.5°; glove change; intra-cavity lavage; wound protection; and closure strategy). The bundle was applied to all consecutive patients undergoing colonic resections. The primary outcome, SSI, was independently assessed by the National Infection Surveillance Committee for up to 30 postoperative days. A historical, institutional pre-implementation control group (2012–2017) with an identical methodology was used for comparison. Findings: In total, 1516 patients were included, of which 1256 (82.8%) were in the control group and 260 (17.2%) were in the post-implementation group. After 2:1 propensity score matching, the groups were similar for all items (*p* > 0.05). Overall compliance with the care bundle was 77% (IQR 77–88). The lowest compliance rates were observed for temperature control (53% overall), intra-cavity lavage (64% overall), and wound protection and closure (68% and 63% in the SSI group, respectively). Surgical site infections were reported in 58 patients (22.2%) vs. 21.4% in the control group (*p* = 0.79). Infection rates were comparable throughout the Centers for Disease Control and Prevention (CDC) categories: superficial, 12 patients (4.5%) vs. 4.2%, *p* = 0.82; deep incisional, 10 patients (3.7%) vs. 5.1%, *p* = 0.34; organ space, 36 (14%) vs. 12.4%, *p* = 0.48. After propensity score matching, rates remained comparable throughout all comparisons (all *p* > 0.05). Conclusions: The implementation of an intraoperative standardized care bundle had no impact on SSI rates. This may be explained by insufficient compliance with the individual measures.

## 1. Introduction

With rates of up to 34%, surgical site infections (SSIs) are frequent complications in colorectal surgery and significantly impact morbidity, the length of hospital stays, and costs [1,2,3]. International guidelines for SSI prevention are available and regularly updated [1,4]. Up to 60% of SSIs may be considered preventable when evidence-based recommendations are applied [5]. Various SSI-preventing bundles have been suggested; however, these have been proposed with different infection-preventing measures [5,6]. The key to the successful implementation of such bundles is compliance with the individual measures. The implementation of new standards of care remains a challenge, and actual compliance in clinical routine is frequently overestimated [7,8,9].

Our group published an institutional series of 1263 patients after colonic surgery between 2012 and 2017 with prospective, independent SSI surveillance [10]. In an attempt to decrease SSI rates, an evidence-based SSI prevention bundle was implemented thereafter. The aim of the present study was to assess compliance with this multimodal care bundle and to evaluate its impact on SSI in colonic resections.

## 2. Methods

### 2.1. Patients and Data Management

Consecutive patients undergoing elective or emergent (surgery during a non-scheduled hospital stay) colonic resections (right, left, segmental, or total colectomy) were prospectively included between 1 November 2018 and 31 October 2020. Revisional or staged procedures were also considered, providing that a colectomy was performed. In this case, the closure algorithm was applied at the time of a revisional or staged procedure when the abdominal cavity was closed, while considering the contamination class at initial exploration. The study was conducted at Lausanne University Hospital (CHUV), a tertiary Swiss referral center. The institutional enhanced recovery after surgery (ERAS) program was implemented in May 2011. According to the pathway, rectal enemas were performed for left and total colectomies, but no oral mechanical bowel preparation was performed [11]. Preoperative oral antibiotics were not administered. Ostomy reversal procedures and rectal procedures were excluded due to a lack of systematic follow-up by the Swiss national infection surveillance system (*Swissnoso*, www.swissnoso.ch (accessed on 1 July 2021)). *Swissnoso* is a nationwide surveillance program of nosocomial infections in Switzerland, according to previously published methodology [12]. This study was approved by the institutional review board (CER-VD # 2020-238).

Demographics, including age and gender; body mass index (BMI) values; American Society of Anesthesiologists (ASA) scores;, and surgical details, including setting (elective vs. emergent), approach (open/converted to open versus laparoscopic), wound contamination class (defined according to the Centers for Disease Control and Prevention (CDC) classification) [13]; and the duration of surgery were prospectively assessed in a dedicated institutional database. Furthermore, National Nosocomial Infections Surveillance (NNIS) scores (0–3) were calculated for each patient based on wound contamination class, their ASA score, and the duration of surgery.

### 2.2. Intervention

The institutional SSI prevention bundle was developed in January 2018, internally validated, and implemented as a standard of care in clinical practice on 1 November 2018. The items in the prevention bundle were identified based on previous work by Cima et al. [14] and validated international guidelines [1,4]. The bundle was composed of 10 evidence-based items with a focus on antibiotic prophylaxis (type, timing (within 60 min) and intraoperative re-dosing), skin disinfection (Chlorhexidin^®^ 2%, B. Braun, Melsungen, Germany), induction and perioperative core temperature control > 36.5° Celsius, glove change before closure, intracavity lavage (not routinely and only in contaminated areas), systematic use of a double-ring wound protection device (Alexis^®^ O-Ring, Applied Medical, Rancho Santa Margarita, CA, USA), and predefined closure strategy. The wound closure strategy was chosen based on the longest skin incision, using an arbitrary cut off at 10 cm to reflect the difference between laparoscopic and open approaches. The details for individual items and closure strategy are provided in Appendix A and Appendix B.

A single-use, closed-wound negative pressure wound therapy (NPWT) device (PICO^®^, Smith and Nephew Inc., Hull, UK) was used for patients without constitutional risk factors and wounds of more than 10 cm with a wound contamination class of II or III. This NPWT device, delivering a constant −80 mmHg of pressure, was removed at postoperative day 5 or the day of discharge, if earlier. For dirty wounds or contaminated wounds in high-risk patients, an open-wound, subcutaneous NPWT device was used (V.A.C.^®^, KCl Inc., San Antonio, TX, USA). This dressing system was changed at postoperative day (POD) 3, and the wound was secondarily closed at POD 6. The surgical staff was periodically educated to manage closed- and open-wound NPWT dressings.

Dedicated checklists were completed by the main surgeon and the anesthetist immediately upon completion of the procedure to assess compliance with individual items (Appendix C). Core temperature was systematically documented on the anesthesia chart and cross-checked with the main surgeon at the end of each procedure).

### 2.3. Outcomes/Study Endpoints

The 30-day SSIs were independently monitored by *Swissnoso*. These were further categorized as superficial incisional, deep incisional, and organ space infections according to the CDC classification [13].

### 2.4. Impact of Covid 19 Pandemic

The COVID-19 pandemic affected the Swiss healthcare system in 2020. Between 1 March 2020 and 31 May 2020, *Swissnoso* decided to temporarily suspend the surveillance program. Furthermore, to relieve pressure on intensive care units, elective surgery for non-oncological indications was suspended from March 2020 up to 31 October 2020 (the end of recruitment) at the Lausanne University Hospital. However, emergency and mandatory oncological resections were maintained to assure essential surgical service for the entire referral region.

### 2.5. Statistical Analysis

Continuous variables were summarized using the median (interquartile range: IQR) or mean ± standard deviation (SD), and categorical variables were summarized using frequencies and percentages. The differences between groups were compared using chi-square tests for categorical variables and Mann–Whitney or independent sample t-tests, as appropriate, for continuous variables.

To overcome bias between the two groups due to the distributions of covariates, a propensity score model was built using multivariate regression analyses to predict the probability of each patient being assigned to either the study or control group based on age, gender, NNIS score, operative approach, setting (elective or emergency), and conversion to open.

The predictive values were then used to build a 2:1 matching, applying the nearest neighbor method with a caliber distance of 0.2.

After matching, the differences between both groups were compared using McNemar tests for categorical variables and Wilcoxon signed-rank tests for continuous variables.

All tests were two-sided and an alpha level of less than 0.05 was considered statistically significant. The analyses were conducted using the Statistical Package for the Social Sciences (SPSS, version 25; SPSS, Inc., Armonk, NY, USA).

## 3. Results

### 3.1. Patients

The study cohort included 1516 patients, of which 1256 (82.8%) were in the control group and 260 (17.2%) were in the post-implementation study group. The demographic and surgical characteristics of both groups are displayed in Table 1.

There were significant differences between the groups regarding gender, surgical setting, contamination class, and composite NNIS scores. After matching, the groups were balanced (Table 2).

### 3.2. Compliance with Care Bundle

**Figure 1:**
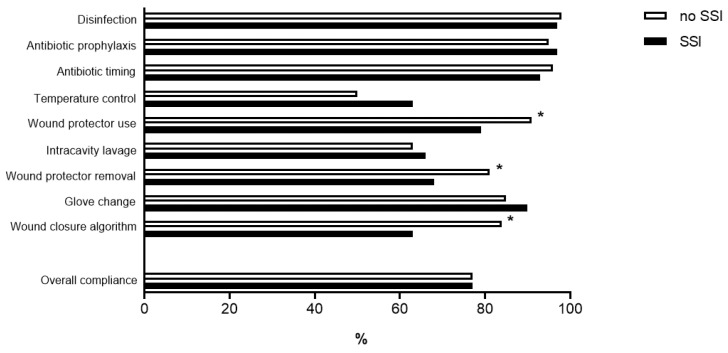
Compliance with the SSI prevention bundle. Compliance in % to individual items of the institutional SSI prevention bundle in patients without SSI (*n* = 202, grey bars) and patients with SSI (*n* = 58, black bars). * indicate statistically significant differences (*p* < 0.05).

The overall level of compliance with the care bundle was 77% (IQR 77–88). The lowest levels of compliance were observed for core temperature control (overall compliance 53%), intra-cavity lavage (overall compliance 64%), and wound protection and closure (68% and 63% in the SSI group, respectively). Figure 1 illustrates individual items of the care bundle and compares patients who developed SSIs to patients who did not. Significant differences regarding wound protection for both device application (79% vs. 91%, *p* = 0.013) and removal (68% vs. 81%, *p* = 0.034) and adherence to the predefined wound closure strategy (63% vs. 84%, *p* < 0.001) were revealed. All other comparisons revealed no statistically significant differences (all *p* > 0.05).

### 3.3. SSIs

**Figure 2:**
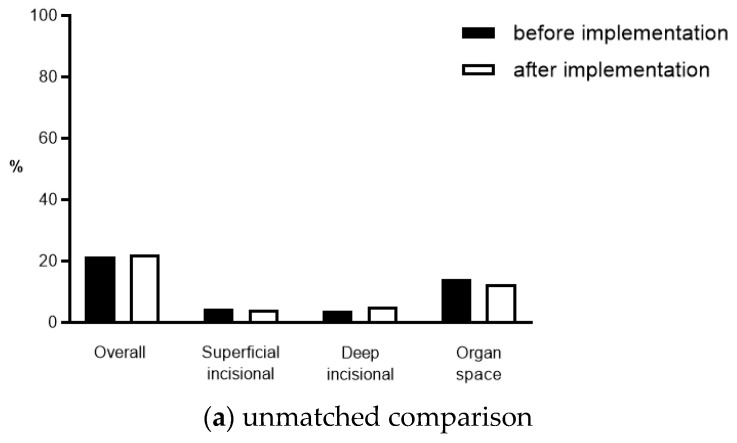

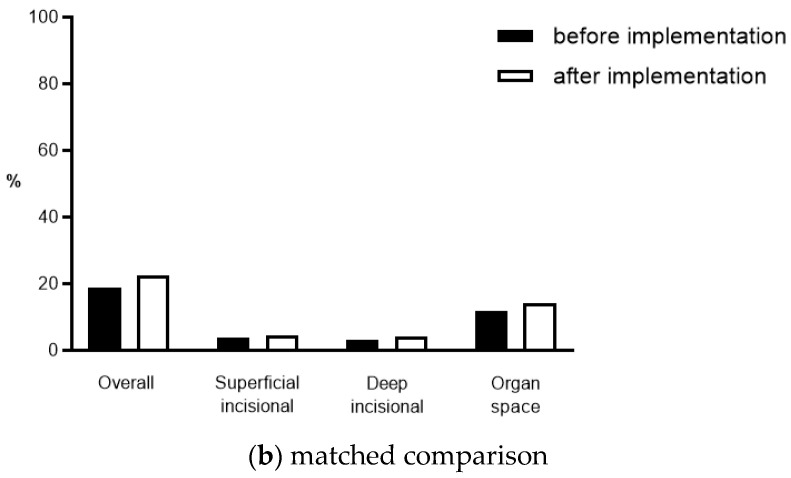
SSI rates. SSI rates before (black bars) and after (grey bars) the implementation of the institutional SSI prevention bundle in (**a**) unmatched and (**b**) propensity score (2:1) matched cohorts. SSI, surgical site infection.

In the unmatched cohort, the overall rates of SSI were 22.2% (58/260 patients) in the post-implementation group vs. 21.4% in the control group (*p* = 0.79). These rates were comparable throughout the CDC categories: superficial incisional, 4.5% vs. 4.2%, *p* = 0.82; deep incisional, 3.7% vs. 5.1%, *p* = 0.34; organ space, 14% vs. 12.4%, *p* = 0.48 (Figure 2a). After propensity score matching, SSI rates remained comparable throughout all comparisons (all *p* > 0.05, Figure 2b).

## 4. Discussion

In this present study, the implementation of a standardized surgical site infection prevention bundle had no beneficial impact on SSI rates as assessed by an independent surveillance organ. This finding may be explained by a lower rate of compliance with the bundle of only 77%, including suboptimal compliance with several critical steps related to wound protection and closure, as demonstrated by the detailed analysis of the individual bundle items.

The evidence-based bundle was launched in 2018 following an initial audit completed between 2012 and 2017 [10]. At a first glance, the results of this institutional quality improvement initiative were disappointing, with overall SSI rates above 20% and no significant impact of the prevention bundle on any of the CDC subcategories. The implementation of new standards of care in a busy clinical practice within a referral center may not be as straightforward as suggested by seemingly flawless practice changes carried out by other centers [6,14,15]. Indeed, former studies reporting on such compliance demonstrated overall rates of around 70%, similar to the results of the present study [16,17]. However, high compliance with all items appears to be the key to success, demonstrating a dose-effect pattern for SSI reduction [17]. Hence, this analysis not only focused on the mere endpoint of SSIs but rather attempted to critically appraise implementation challenges through a detailed analysis of individual items.

The rather high SSI rate of over 20% is comparable to results from former national surveillance studies that employed a similar methodology [12,18]. Of note, published rates are highly dependent on the quality of surveillance, which may contribute to the large variation in SSI rates described in the literature. Therefore, methods of prospective and independent surveillance may lead to higher SSI rates. Surprisingly, the critical assessment of our institutional practice and the substantial efforts to implement an evidence-based care bundle did not translate into a clinical benefit.

Three main areas of improvement were revealed. The first was related to the routinely applied wound protection device. Both the omission *and* incorrect removal (*after* the intra-cavity lavage but *before* glove change and the switch to the closing tray) of the protection device may have been SSI triggers given the significant difference between patients with and without SSIs (Figure 1).

Correct wound protection may be neglected during a challenging surgical procedure or may simply be deemed unnecessary. However, despite some conflicting results, several contemporary studies have demonstrated the beneficial impact of a circumferential double-ring wound retractor device on incisional SSI, provided that it is properly applied [19,20,21].

Second, low compliance with the standardized wound closure algorithm was noted. This algorithm was tailored to the extent of the surgical incision, perioperative contamination, and constitutional risk factors. A cautious algorithm with a low threshold was chosen to apply either closed- or open-wound NPWT devices. These devices have shown some benefit across various surgical specialties [22]. These results also apply to colorectal surgery; however, when focusing on randomized control trials, the results are conflicting [23,24,25]. The rather low compliance rate of around 60% in patients with SSI reflects the suboptimal application of this algorithm, despite internal development, validation, and staff education.

Third, the present study revealed low overall adherence to perioperative “core temperature” control. The detailed assessment of this item through repeated measurements by the anesthesia team during the procedure identified insufficient compliance with this critical item [26,27]. General anesthetics substantially impair thermoregulation, synchronously reducing the thresholds for vasoconstriction and shivering [28]. Consequently, unwarmed anesthetized patients become hypothermic by 1–2 °C. The direct association of perioperative hypothermia and SSI has been repeatedly demonstrated, and the findings of the present study emphasize the importance of perioperative temperature monitoring with hypothermia prevention measures.

In order to improve compliance, the results of this present study were presented to institutional surgical and anesthesia staff in dedicated training sessions. Whether improved compliance can be achieved needs to be further evaluated through an institutional audit.

This study has several limitations. First, data for compliance with the prevention bundle items for the pre-implementation period were not available and thus impede further comparison. Second, some recommendations, such as antibiotic prophylaxis and the timing of administration, were already followed through institutional guidelines, while others (i.e., wound protector use, intracavity lavage, and closure strategy) relied on the discretion of individual surgeons. Third, the available data points, and thus potential confounding variables, were limited given the focus on readily available items by the independent surveillance committee. However, both study periods were subject to identical methodology with prospective data gathering in consecutive patients, thus limiting the risk of inter-observer bias. Finally, the COVID-19 pandemic probably introduced bias through suspended surveillance periods, patient selection or prioritization, and loss to follow-up. Propensity score matching was chosen to statistically adjust for imbalances between the groups.

In conclusion, the quality improvement effort of introducing a standardized, evidence-based, multimodal intraoperative infection prevention bundle did not result in decreased SSI rates according to these preliminary results. This may be explained by the overall compliance rate of 77%, which may be insufficient. It remains to be explored whether increased compliance with individual measures, such as wound protection, closure strategies, and better core temperature control, could induce a clinical benefit.

## Figures and Tables

**Table 1:** Demographic characteristics and surgical details.

	Pre-Implementation *n* = 1256	Post-Implementation *n* = 260	Total *n* = 1516	*p*-Value
Age, median (IQR)	67 (53–76)	65 (53–75)	66 (53–76)	0.588
Male gender (%)	667 (53.1)	156 (60)	823 (54.3)	0.041
ASA class ≥ 3 (%)	556 (44.3)	114 (43.8)	670 (44.2)	0.901
BMI (kg/m^2^, mean ± SD)	24.7 ± 5.5	25.1 ± 5.3	24.9 ± 5.4	0.427
Emergency (%)	530 (42.2)	85 (32.7)	615 (40.6)	0.004
Laparoscopy (%)	729 (58.0)	136 (52.3)	865 (57.1)	0.09
Conversion (%)	187 (14.9)	29 (11.1)	216 (14.2)	0.107
Surgical duration (min, mean ± SD)	170 ± 100	172 ± 102	170 ± 100	0.857
Contamination class (%)				<0.001
II	566 (45.1)	156 (60)	722 (47.6)	
III	388 (30.9)	32 (12.3)	420 (27.7)	
IV	302 (24.0)	72 (27.7)	374 (24.7)	
NNIS score (%)				<0.001
0	188 (15.0)	74 (28.5)	262 (17.3)	
1	455 (36.2)	97 (37.3)	552 (36.4)	
2	484 (38.5)	79 (30.4)	563 (37.1)	
3	129 (10.3)	10 (3.8)	139 (9.2)	

Baseline demographic parameters of patients before and after implementation of the SSI prevention bundle. ASA, American Society of Anesthesiology; BMI, body mass index; SD, standard deviation; NNIS, National Nosocomial Infection Surveillance (including ASA score, wound contamination class, and surgical duration). Age is presented as the median and interquartile range (IQR); BMI and surgical duration are presented as mean ± standard deviation (SD). All other variables are reported as frequencies with percentages. Bolded *p*-values indicate statistical significance (*p* < 0.05).

**Table 2:** Demographic characteristics and surgical details after matching.

	Pre-Implementation *n* = 520	Post-Implementation *n* = 260	Total *n* = 780	*p*-Value McNemar
Age, median (IQR)	66 (5–75)	65 (53–75)	66 (54–75)	0.706 *
Male gender (%)	305 (58.7)	156 (60)	461 (59.1)	0.928
Emergency (%)	164 (31.5)	85 (32.7)	249 (31.9)	>0.99
Laparoscopy (%)	279 (53.7)	136 (52.3)	415 (53.2)	>0.99
Conversion (%)	49 (17.6)	29 (21.3)	78 (18.8)	0.542
NNIS score (%)				0.402
0	131 (25.2)	74 (28.5)	205 (26.3)	
1	227 (43.7)	97 (37.3)	324 (41.5)	
2	139 (26.7)	79 (30.4)	218 (27.9)	
3	23 (4.4)	10 (3.8)	33 (4.2)	

Baseline demographic parameters of patients before and after implementation of the SSI prevention bundle and after 2:1 propensity score matching. NNIS, National Nosocomial Infection Surveillance (including ASA score, wound contamination class, and surgical duration). * Wilcoxon signed-rank test results.

## Data Availability

The data of this institutional quality improvement initiative is not publicly available. The authors can be contacted for data-related queries.

## Appendix A. Core Measures

(1) Disinfection	Chlorhexidine WAIT UNTIL DRY!
(2) Antibiotic prophylaxis	Cefuroxime 1.5 g + metronidazole 500 mg (or according to protocol if contra-indication) Within 60 min of incision (upon start of anesthesia) Preexisting antibiotic treatment: Redosing within 60 min of incision
(3) Prevent hypothermia	Maintain body temperature of at least 36.5 °C (documented every 30 min) using a forced-air warming device Induction of anesthesia should not begin unless the patient’s temperature is >36 °C (unless clinical urgency) Ambient temperature of at least 21 °C Intravenous fluids and blood products warmed to 37 °C Irrigation fluids should be warmed to 38–40 °C
(4) Wound protection	Systematic use of wound protector (Alexis) Remove Alexis after intra-cavity lavage (if performed) but BEFORE use of closing tray
(5) Intracavity lavage	Not routinely (no dilution of host defense) Lavage only in contaminated areas (i.e., pelvis), consider use of gauze instead If complete lavage performed (III–IV): use saline leave drain until patient mobilized for 24 h *or* consider 2nd look (algorithm staged procedure)
(6) Use of closing tray	Before fascia closure/after intra-cavity lavage (if performed): GLOVE CHANGE Systematic reminder by scrub nurse
(7) Appropriate closure strategy (see Appendix B)	

## Appendix B. Wound Closure Strategy

BMI, body mass index; OP, operation; PICO: single-use negative pressure device; VAC, vacuum-assisted closure. Contamination class is classified according to the Centers for Disease Control and Prevention.

## Appendix C. Checklist

Checklist of the SSI prevention bundle. This checklist was completed by the main surgeon at the end of the procedure. CI, contraindication; Alexis, O-ring wound protector device; PICO, single-use negative pressure wound therapy device; VAC, vacuum-assisted closure; ttt, treatment. Contamination class is classified according to the Centers for Disease Control and Prevention.
